# Identification of QTLs associated with tissue culture response of mature wheat embryos

**DOI:** 10.1186/s40064-016-3241-y

**Published:** 2016-09-13

**Authors:** Jian Ma, Mei Deng, Si-Yu Lv, Qiang Yang, Qian-Tao Jiang, Peng-Fei Qi, Wei Li, Guo-Yue Chen, Xiu-Jin Lan, Yu-Ming Wei

**Affiliations:** Triticeae Research Institute, Sichuan Agricultural University, 211 Huimin Road, Wenjiang, Chengdu, 611130 Sichuan China

**Keywords:** Wheat, Callus rate, Differentiation, Plant regeneration rate, QTL

## Abstract

Mature embryo is an excellent explant for tissue culture as it is convenient to be obtained without limitation of growing seasons and development stages. However, regeneration ability of the calli from wheat mature embryos is limited, thus hindering its application. To identify genes associated with the tissue culture response (TCR) of wheat, QTLs for callus induction from mature embryos and callus regeneration were detected using a recombinant inbred lines (RILs) population derived from the cross between a synthetic hexaploid wheat genotype, SHW-L1 and a commercial cultivar Chuanmai 32. Three QTLs for callus rate were identified and they were located on chromosomes 1D, 5A, and 6D, respectively, with explained phenotypic variation ranging from 10.16 to 11.82 %. One QTL for differentiation rate was detected only with 10.96 % of the phenotypic variation explained. Two QTLs for emergence rate were identified and they were located on 3B and 4A, respectively, with 9.88 and 10.30 % of phenotypic variation. The results presented in this study with those reported previously indicated that group 1, 3, and 5 chromosomes are likely to play important roles in TCR of wheat.

## Background

Gene engineering technique is an important tool for functional studies of a given gene and genetic modification of cereal crops. An excellent tissue culture system is the prerequisite of highly efficient genetic transformation. Immature embryo is recognized as an excellent explant for wheat genetic transformation (Yin et al. 2011). However, obtaining immature embryos is limited by the growth season and controlled conditions, thus affecting the progress of tissue culture and therefore gene transformation (Medvecká and Harwood 2015). Thus, an alternative explant replacing immature embryos is favored. The mature embryo is such one of these alternatives as it is convenient to be obtained without limitation of growing seasons and development stages (Yin et al. 2011). Nonetheless, it is reported that the regeneration ability of the calli from mature embryos of wheat is limited, thus hindering its application. Numerous studies have focused on improving regeneration ability by adjusting constitutes and concentration of hormones (Gill et al. 2014; Li et al. 2013b; Murín et al. 2012; Shah et al. 2003; Turhan and Baser 2004), using whole explant culture, endosperm supported culture, thin pieces culture and explants cutting culture (Tao et al. 2008). It is reported that genotypes of the donors greatly affect the efficiency of callus induction since no culture conditions have been reported suitable for all genotypes (Bolibok and Rakoczy-Trojanowska 2006; Ge et al. 2006).

It is believed that genetic control of plant regeneration ability could be either qualitative (Reisch and Bingham 1980) or quantitative (Taguchi-Shiobara et al. 2006). A large number of studies have been reported on quantitative trait loci (QTLs) analysis for tissue culture response (TCR) in soybean (Yang et al. 2011), rice (Li et al. 2013a; Nishimura et al. 2005; Zhao et al. 2009), maize (Murigneux et al. 1994; Wan et al. 1992), barley (Bregitzer and Campbell 2001; Manninen 2000; Mano and Komatsuda 2002), rye (Bolibok et al. 2007), and wheat (Jia et al. 2007; Nielsen et al. 2015; Torp et al. 2001).

With introduced D genome of *Aegilops tauschii*, synthetic hexaploid wheat (SHW) could offer breeder novel genetic variations (Mares and Mrva 2008). It is widely accepted that SHW lines are superior in disease resistance, abiotic stress tolerance, grain quality, and tolerance to pre-harvest sprouting (Lage et al. 2003; Trethowan and Mujeeb-Kazi 2008; Yang et al. 2009). A full understanding of the genetic basis for these desirable traits is important in exploring favored genes/locus. The present study is one of such efforts aiming to identify important genes. Diversity Arrays Technology (DArT) is a rapid and DNA sequence-independent technique and can detect and type DNA variation at several hundred genomic loci in parallel (Jaccoud et al. 2001; Wenzl et al. 2004).

Compared with those in rice, fewer QTLs controlling TCR of mature embryo have been reported and our knowledge on the genetic basis of TCR in bread wheat is still limited. In this study, we are aiming at performing whole-genome linkage mapping using DArT markers to identify QTLs associated with callus induction from mature embryos and plantlet regeneration from calli using a population of recombinant inbred lines (RILs) derived from a cross between a synthetic hexaploid wheat (SHW-L1) and a cultivar Chuanmai 32.

## Methods

### Plant materials

A population of 171 F8 recombinant inbred lines (RILs) derived by single-seed descent from the F2 of SHW-L1/Chuanmai 32 was used in this study. SHW-L1 is a line of SHW crossed between the *Triticum. turgidum* ssp*. turgidum* AS2255 (AABB) and *Ae. tauschii* ssp*. tauschii* AS60 (Zhang et al. 2004). Chuanmai 32 is a commercial cultivar of hexaploid wheat grown in southwest winter wheat areas of China. Emergence rate of SHW-L1 was significantly higher than that of Chuanmai32.

The F8 to F10 RILs were planted in the experimental farm of the Triticeae Research Institute, Sichuan Agricultural University, China in 2011–2012, 2012–2013, and 2013–2014, respectively. Each line was single-seed planted in two 2 m long rows, with 30 cm between rows and 10 cm between plants within rows. Field management followed common practices for wheat production. Embryo isolation of the seeds was conducted 1 year later after the seeds had been harvested.

### Callus induction

Callus induction and regeneration were performed using methods described by Jia et al. (2007) with some modifications. Mature seeds were first sterilized in 75 % ethanol for 5 min and then in 25 % (v/v) sodium hypochlorite solution for 25 min. They were then immersed in sterile distilled water for 12 h, sterilized again in 25 % (v/v) sodium hypochlorite solution for 25 min and washed 4 times with sterile distilled water after each sterilization. The embryos isolated (wiped off embryonic tips) from the imbibed seeds were placed scutellum down on solid MS medium (pH = 5.8) containing MS basal salts and B1 vitamins supplemented with 2.5 mg L^−1^ 2,4-D, 30 g L^−1^ maltose and 1 g L^−1^ casein hydrolysate. Induction was performed in the dark with 30 embryos per petri dish. All the RILs were cultured and the petri dishes were placed in a culture room at a room temperature (RT) of 25 °C. Initially, 90 or more embryos from each line were cultured. Another 30–90 embryos from selected lines were cultured to make up for loss from contamination. The condition of culture was strictly kept uniformly to reduce experimental errors. Embryos forming calli were scored after 28 days of induction. The percentage of embryos forming a callus (callus rate) was used to estimate callus induction efficiency.

### Regeneration

Following 28 day of induction intact calli were used for regeneration induction. They were transferred to regeneration induction medium containing 1 μM IAA and 160 mg L^−1^ timentin. Culture was conducted at RT in the culture room with a daily cycle of 16 h light and 8 h dark. The fraction of calli that formed green spots and the fraction of regeneration calli were counted 28 day after regeneration induction. Percent calli forming green spots (differentiation rate) and percent calli regenerating plants (emergence rate) were used to estimate regeneration potential and capacity, respectively.

### Statistical analysis and QTL mapping

Pearson correlation analysis and Shapiro-Wilka statistical test (W test) were conducted using IBM SPSS Statistics 19. The existing linkage map for the population used in this study, which has a total length of 3766.9 cM and contains 1862 loci (1794 Diversity Arrays Technology (DArT) and 68 simple sequence repeat markers) with an average distance of 2.0 cM between markers (Yu et al. 2015) was used for QTL mapping. QTL analysis was conducted using QTL IciMapping version 3.2 following inclusive composite interval mapping (ICIM) (Li et al. 2007). The walking speed for all QTLs was 1.0 cM, and the logarithm of odds score threshold was set at 2.5 (Lin et al. 1996). QTLs were named according to International Rules of Genetic Nomenclature (http://wheat.pw.usda.gov/ggpages/wgc/98/Intro.htm).

## Results and discussion

**Fig. 1:**
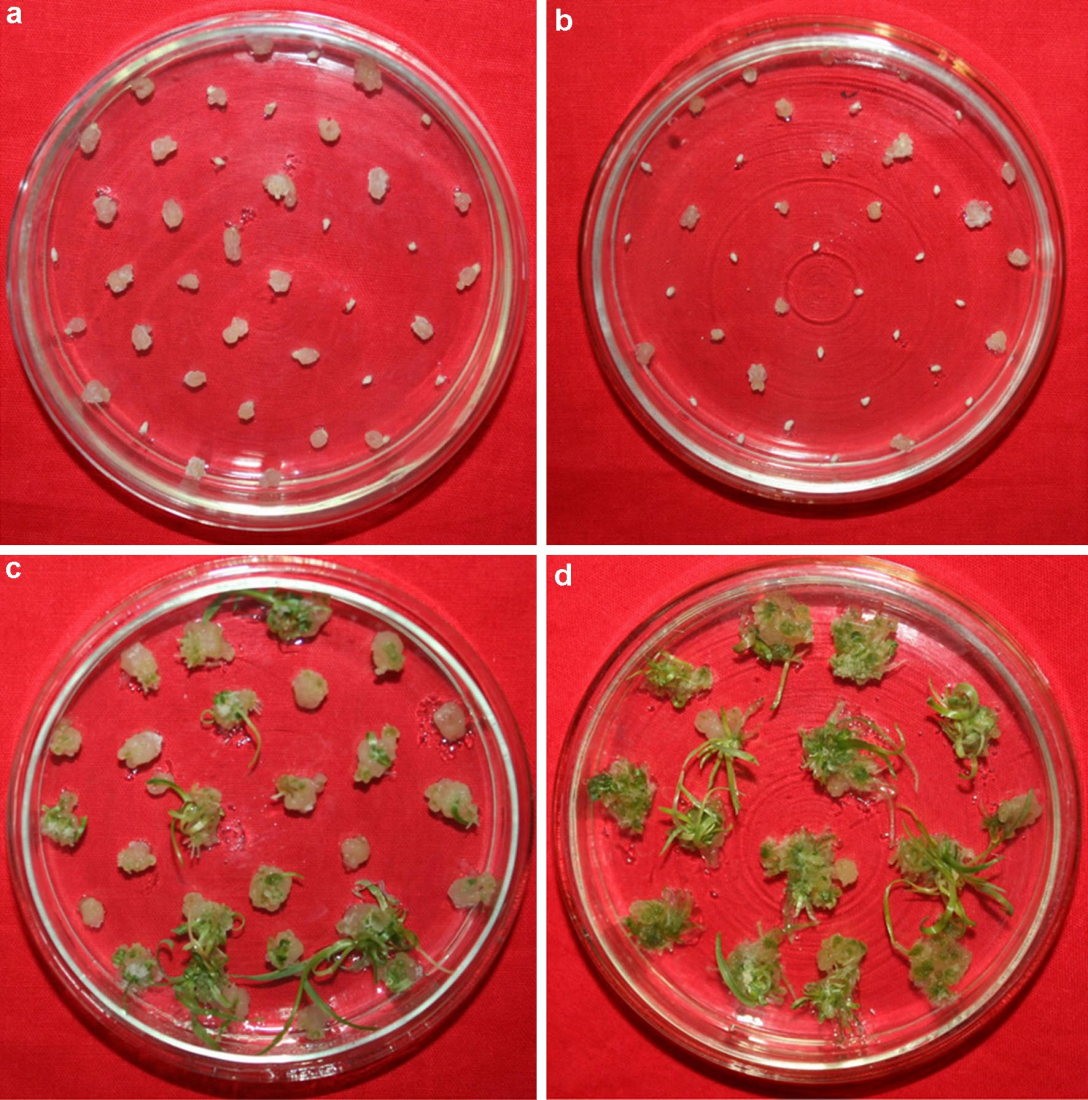
Tissue culture response of mature embryos. **a**, **b** Callus induction of Chuanmai32 and SHW-L1, respectively; **c**, **d** the difference of callus regeneration between Chuanmai32 and SHW-L1

**Fig. 2:**
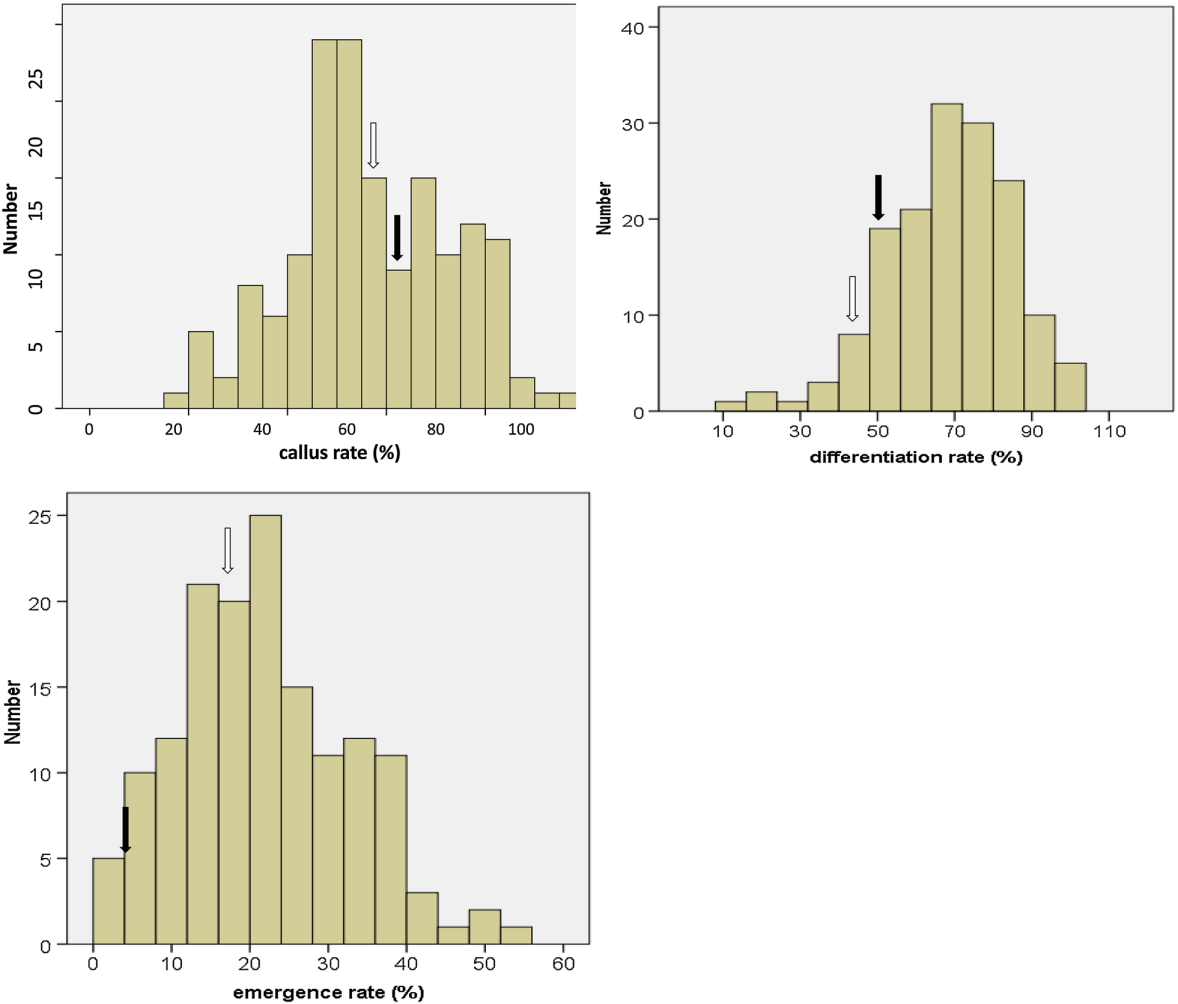
Frequency distributions of the three traits from the RIL population Data was based on average over the investigated experiments. The *black arrow* indicates the value of SHW-L1 and the *blank arrow* indicates Chuanmai 32

Significant differences were not detected for callus rate between the two parents, SHW-L1 and Chuanmai 32 (Fig. 1). However, callus rate varied greatly among the RILs and showed a continuous distribution (*p* = 0.196 for test of normality) ranging from 12.2 to 96.2 % with a mean of 56.4 % (Table 1; Fig. 2). Significant difference in differentiation rate was also not detected between the two parents. The differentiation rate of the RIL population gave a continuous distribution (*p* = 0.413 for test of normality) with a range between 19.1 and 95.0 % with an average value of 68.1 %. For emergence rate, significant difference between parents was detected with SHW-L1 (17. 4 %) significantly higher than Chuanmai 32 (4.1 %) (Fig. 1). Emergence rate of the population (*p* = 0.061 for test of normality) ranges from 2.1 to 73.3 % with an average of 21.3 % (Table 1; Fig. 2). Pearson correlation analysis showed that callus rate was significantly and positively correlated with differentiation rate; and differentiation rate was significantly and positively correlated with emergence rate (Table 2).

**Table 1:** Parameters (%) of tissue culture response traits in RIL population

Trait	Parents^a^		2012^b^		2013^b^		2014^b^		Average^c^	
	SHW-L1	Chuanmai 32	Mean	Range	Mean	Range	Mean	Range	Mean	Range
Callus rate	59.3	62.2	57.8	14.2–92.5	48.9	4.5–99.0	62.4	18.0–97.0	56.4	12.2–96.2
Differentiation rate	43.2	47.3	75.4	16.3–100.0	39.3	3.3–85.0	89.4	37.5–100.0	68.1	19.1–95.0
Emergence rate	17.4**	4.1	13.3	0.0–70.0	30	0.0–80.0	–	–	21.3	2.1–73.3

** Significant differences between SHW-L1 and Chuanmai 32 at *p* = 0.01

^a^The values were means of data from 3 or 2 years

^b^The values were calculated from the data of the 171 lines of the population

^c^The average was calculated by the data of 2 or 3 years

**Table 2:** Correlation analysis of the three investigated traits in RIL population

	Callus rate	Differentiation rate	Emergence rate
Callus rate			
Pearson correlation	1	0.315**	0.066
Significance (two-tailed)		<0.01	0.43
# of analyzed lines	149	149	149
Differentiation rate			
Pearson correlation	0.315**	1	0.318**
Significance (two-tailed)	<0.01		<0.01
# of analyzed lines	149	149	149
Emergence rate			
Pearson correlation	0.066	0.318**	1
Significance (two-tailed)	0.43	<0.01	
# of analyzed lines	149	149	149

**Significant differences at *p* = 0.01

**Fig. 3:**
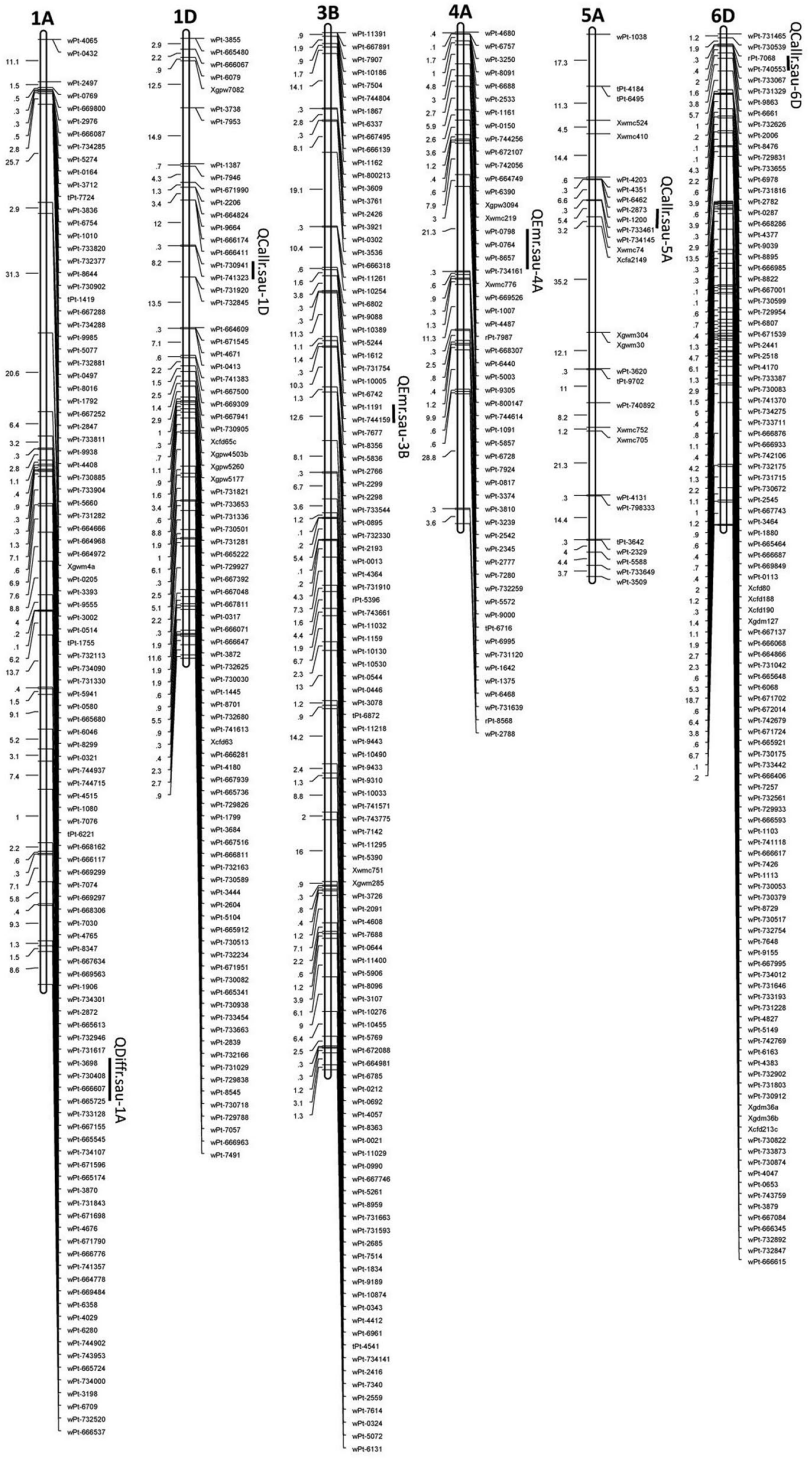
QTLs for the three traits of the RIL population. Map distances (cM) are indicated on the *left* of each chromosomes, and markers names are on the* right*

Three QTLs for callus rate were identified in each of the three seasons assessed. They were located on chromosomes 1D, 5A, and 6D, respectively (Table 3; Fig. 3). Each of these loci explained between 10.16 and 11.82 % of the phenotypic variation. Positive alleles of these QTLs were derived from Chuanmai 32 (Table 3). A single QTL, *QDiffr.sau*-*1A*, was detected for differentiation rate only in a single growing season. The phenotypic variation explained by this QTL was 10.96 %. Positive allele of this locus was derived from SHW-L1. Two QTLs for emergence rate were identified in a single growing season and they were located on 3B and 4A, respectively. The positive alleles of these QTLs were derived from Chuanmai 32. The phenotypic variation for these QTLs was 9.88 and 10.30 %, respectively (Table 3).

**Table 3:** QTL controlling callus rate, differentiation rate, and emergence rate detected in the RIL population derived from the cross of SHW-L1/Chuanmai 32

Trait	QTL	Chromo-some	Trail	Position	Left marker^a^	Right marker^a^	LOD^b^	PVE (%)^c^	Add^d^
Callus rate	*QCallr.sau*-*6D*	6D	2012	1.21	wPt-730539	rPt-7068	3.88	11.44	−0.07
	*QCallr.sau*-*5A*	5A	2013	54.41	wPt-2873/wPt-1200	wPt-733461	3.84	10.16	−0.09
	*QCallr.sau*-*1D*	1D	2014	55.11	wPt-730941	wPt-741323	5.02	11.82	−0.07
Differentiation rate	*QDiffr.sau*-*1A*	1A	2012	180.71	wPt-3698/wPt-730408	wPt-666607/wPt-665725	3.96	10.96	0.07
Emergence rate	*QEmr.sau*-*3B*	3B	2014	78.71	wPt-1191	wPt-744159	3.50	9.88	−0.05
	*QEmr.sau*-*4A*	4A	2014	21.61	wPt-0798	wPt-0764/wPt-8657/wPt-734161	3.50	10.3	−0.06

^a^The left and right flanking marker of the interval of LOD peak value for a given QTL

^b^The logarithm of odds

^c^Percentage of the phenotype variation explained

^d^Additive effect at QTL, positive values indicate the effect from SHW-L1, negative values indicate the effect from Chuanmai 32

As indicated in the study of Jia et al. (2007), group 5 chromosomes are likely to play key roles in TCR of *Triticeae* crops. Here, a QTL *QCallr.sau*-*5A* for callus rate was identified. Previous studies identified QTLs for percentage of embryos forming a callus (PEFC) and percentage of calli regenerating plantlets (PCRP) on 5A and 5B chromosomes during culturing of both mature and immature wheat embryos (Jia et al. 2007, 2009). In addition, association analysis identified loci on 5A (wPt-6135) and 5B (tPt-4184) associated with embryo formation in culturing of wheat microspores (Nielsen et al. 2015). In the culturing of barley immature embryo, a QTL controlling callus growth was also identified on 5H in barley (Mano and Komatsuda 2002).

In correspondence to *QCallr.sau*-*1D* for callus rate and *QDiffr.sau*-*1A* for differentiation rate detected on 1D and 1A in wheat, a QTL controlling shoot differentiation ratio was identified on 1H chromosome in barley (Mano and Komatsuda 2002). Nielsen et al. (2015) identified a locus on 1B which was associated with albino and green plantlet regeneration. Different from QTLs for PCRP in wheat mature and immature embryo culture located on 3A (Jia et al. 2007, 2009), a QTL QEmr.sau-3B for emergence rate was identified on 3B. A QTL controlling shoot differentiation ratio was detected on 3H chromosome for immature embryo culture in barley (Mano and Komatsuda 2002). These results indicated that both group 1 and group 3 chromosomes of *Triticeae* crops could be important in TCR.

In addition to those QTLs on group 1, 3, and 5 chromosomes, two QTLs for callus and emergence rates were identified on located on 6D and 4A, respectively. The fact that very few QTLs for TCR in wheat were identified on 6D and 4A indicates that these two QTLs could be novel loci.

Some QTLs described in this study, were detected in one environment only, indicating that TCR is dependent on not only genotype but also environment. This result is in accordance with the study showing that green plantlet formation is highly dependent on both environment and genotype during wheat microspore culture (Holme et al. 1999). Thus, it is recommended that the wheat should be planted in controlled environments such as glasshouse for harvesting mature seeds for tissue culture.

## Conclusions

Here, we report the identification of QTLs for callus induction and callus regeneration from mature embryos based on a RIL population. Three QTLs for callus rate were identified and they were located on chromosomes 1D, 5A, and 6D, respectively, and they explained phenotypic variation ranging from 10.16 to 11.82 %. One QTL for differentiation rate was detected only with 10.96 % of the phenotypic variation explained. Two QTLs for emergence rate were identified and they were located on 3B and 4A, respectively, with 9.88 and 10.30 % of phenotypic variation.
